# Venlafaxine-induced serotonin syndrome causing bilateral cerebral strokes: a case report

**DOI:** 10.3389/fstro.2024.1529674

**Published:** 2025-01-13

**Authors:** Nils Mein, Khadija Mammadli, Felix Luessi, Timo Uphaus

**Affiliations:** ^1^Section of Translational Neuroimmunology, Department of Neurology, Jena University Hospital, Jena, Germany; ^2^Department of Neurology, University Medical Center of the Johannes Gutenberg University Mainz, Mainz, Germany; ^3^Department of Neurology, Focus Program Translational Neuroscience (FTN) and Immunotherapy (FZI), Rhine Main Neuroscience Network (rmn2), University Medical Center of the Johannes Gutenberg University Mainz, Mainz, Germany

**Keywords:** venlafaxine, serotonin syndrome, ischemic stroke, vasospasm, cerebral injury

## Abstract

A 21-year-old Caucasian woman was admitted to our neurologic intermediate care unit after attempting suicide by ingesting an estimated 15 g venlafaxine (Trevilor retard^®^), adding up to a serum concentration of approximately 17,943 μg/l. Brain magnetic resonance imaging (MRI) revealed bilateral cortical restricted-diffusion patterns, indicating ischemic lesions. We report a case of venlafaxine-induced serotonin syndrome most likely cumulating in diffuse artery vasospasm due to an autonomic effect mediated by the serotonergic and adrenergic systems, causing myocardial and cerebral injuries. The serotonin syndrome was treated symptomatically by administering fluids and benzodiazepines and managing the hyperthermia using paracetamol; also, medication with venlafaxine was stopped, and the hypoglycemia was treated. After 6 days, our patient was discharged to the psychiatric facility with no remaining neurologic deficit. The case report provides evidence of ischemic stroke as a rare adverse event of venlafaxine intoxication. Furthermore, we aim to increase awareness of hypoglycemia and epileptic seizures as complications of venlafaxine intoxication. In addition, we demonstrate important pitfalls in the diagnostic procedure and propose a treatment regimen for the underlying serotonin syndrome.

## Introduction

As a serotonin–norepinephrine–dopamine reuptake inhibitor, venlafaxine works as a bicyclic drug and is often used for treating depression, general anxiety disorder, phobic disorder, panic disorder, and, as in this case of off-label use, borderline personality disorder by affecting the serotonin and norepinephrine concentrations in the central nervous system (Holliday and Benfield, 1995; Kent, 2000). For venlafaxine, no dose escalation study, identifying optimal efficacy and tolerability, has been performed so far (Kent, 2000; Hieronymus, 2019). This makes patients using venlafaxine treatment, especially those with increased risk of dying by suicide or suicide attempts in the past, susceptible to intoxication or overdosage, as can be seen in this case report. In the case of venlafaxine intoxication, prolonged, glucose-resistant hypoglycemia and acute myocardial injury due to coronary artery vasospasm have been described (Caroselli and Ricci, 2010; Özdemir, 2022). Commonly described neurologic adverse events following venlafaxine intoxication include seizures and impaired consciousness (Khalifa and Daleau, 1999).

## Case description

Here, we report the case of a 21-year-old Caucasian woman with a history of borderline personality disorder who was admitted to our neurologic intermediate care unit following venlafaxine intoxication. A borderline personality disorder diagnosis was made 10 years ago, and since then, 150 mg of venlafaxine (Trevilor retard^®^) once daily has been prescribed. Apart from that, our patient did not suffer from any additional chronic disease, and no further medication was taken daily. Anamnestic evidence of another suicide attempt dating back 8 years was found. The patient presented with a negative family history of cardiovascular events or psychiatric morbidities. No relevant past interventions were mentioned, and there was no prior admission to the clinical facility.

**Figure 1:**
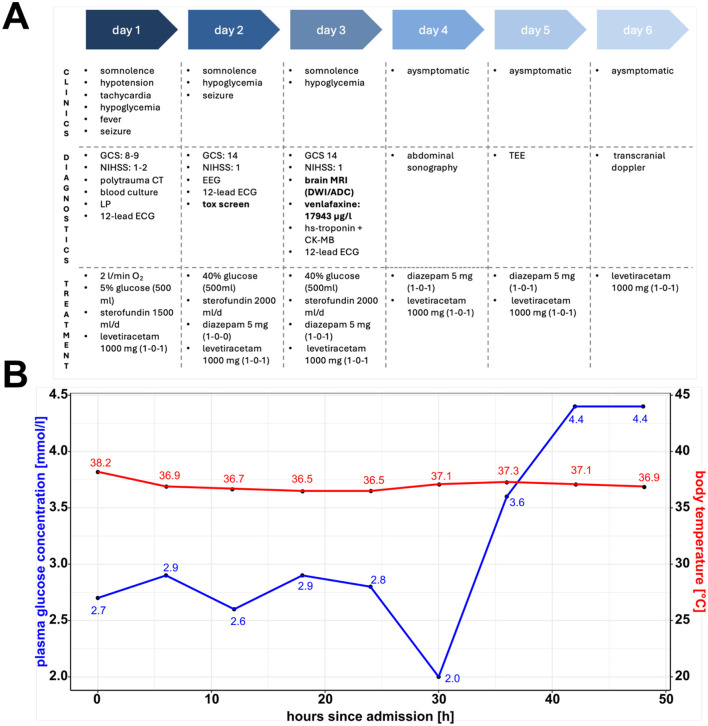
Clinical timeline, plasma glucose concentration and body temperature. **(A)** Clinical timeline showcasing symptoms, diagnostic, and treatment procedures during the patient's hospitalization. Highlighted are the toxicology screen results and brain magnetic resonance imaging scan, which led to the working hypothesis of a venlafaxine-induced serotonin syndrome cumulating in cerebral injury. **(B)** Plasma glucose concentration and body temperature over time highlighting features of the serotonin syndrome. GCS, Glasgow Coma Scale; NIHSS, National Institutes of Health Stroke Scale; LP, lumbar puncture; EEG, electroencephalogram; ECG, electrocardiogram; tox, toxicology; MRI, magnetic resonance imaging; DWI, diffusion-weighted imaging; ACD, apparent diffusion coefficient; TEE, transesophageal echocardiography. **(A)** Was created with BioRender.com. **(B)** Was created using R (1.4.1717) and R Studio (2021.09.0).

On admission (Figures 1A, B), the patient presented with an impaired state of consciousness, with a Glasgow Coma Scale score of 8; severe hypoglycemia of 2.7 mmol/l, hyperthermia (38.2°C); and an arterial blood oxygen saturation of 91%, requiring an O_2_ flow of 2 l/min. Her vital parameters were unstable, with systolic blood pressure ranging between 70 and 100 mmHg and tachycardia of 150 bpm. During the first 3 days, our patient had several focal epileptic seizures with tonic-clonic contractions of both arms, presenting the Figure 4 sign. However, no epileptic potentials were registered in the interictal electroencephalogram. During the first 2 days, our patient's state of consciousness did not adequately improve; she continued having epileptic seizures and glucose-resistant hypoglycemia. Two days after admission, we found out about her prescribed medication of venlafaxine (Trevilor retard) 150 mg once daily through external anamnesis. On day 2, a toxicology screening (cocaine, amphetamine, benzodiazepine, cannabinoids, opiate, methadone, methamphetamine, and venlafaxine) was performed, revealing a serum concentration of venlafaxine of 17,943 μg/l (normal range: 100–400 μg/l) on day 3 (incidentally positive for cannabinoids), which led to our working hypothesis of a serotonin syndrome caused by venlafaxine intoxication. According to our patient, she took an overdose of extended-release venlafaxine hydrochloride 150 mg, an estimated amount of 15 g, in a suicide attempt.

**Figure 2:**
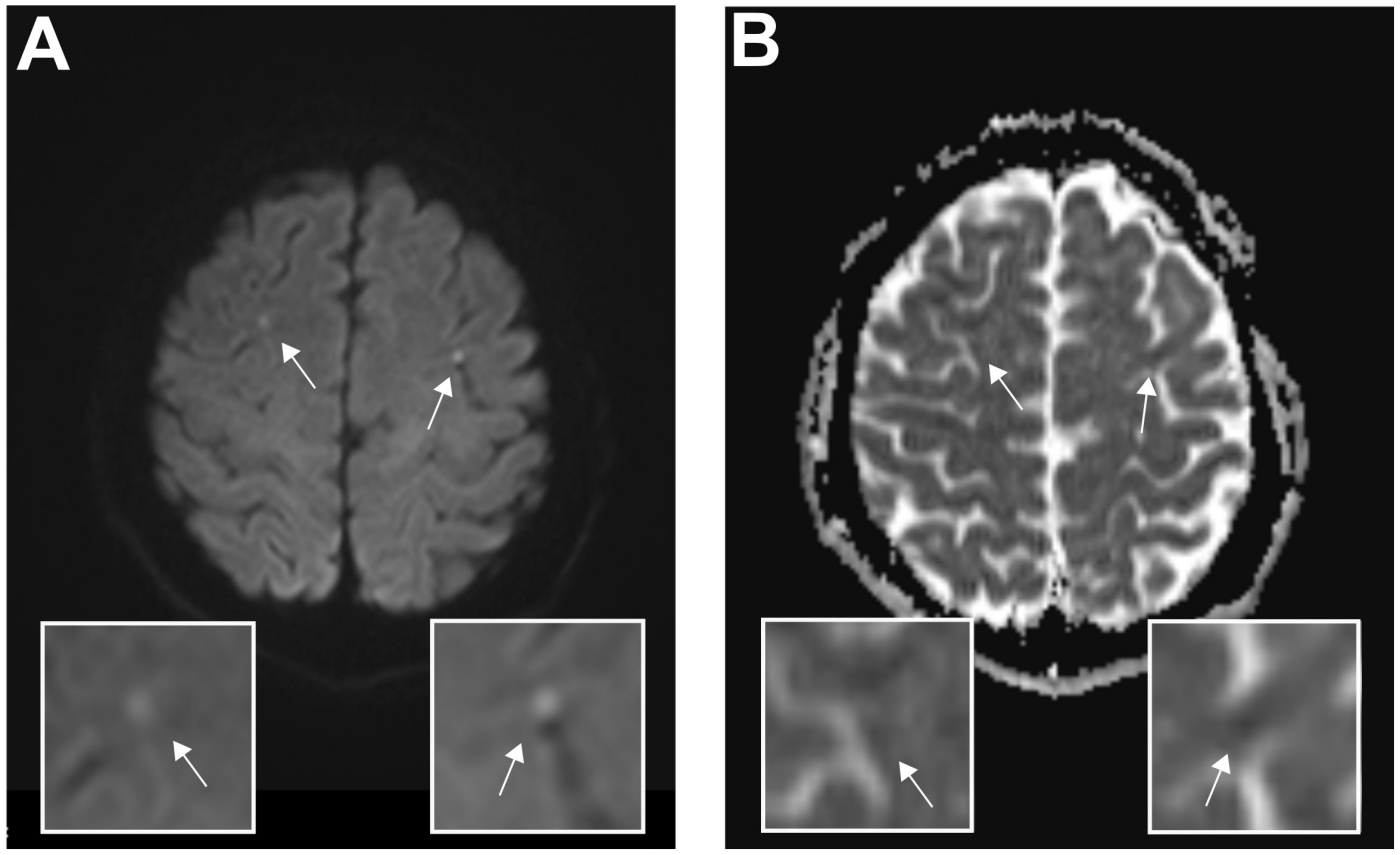
Magnetic resonance imaging reveals bilateral diffusion restrictions indicating ischemic lesions. Axial cerebral magnetic resonance imaging of the acute cerebral infarction post–venlafaxine intoxication on day 4 after admission. **(A)** Diffusion-weighted magnetic resonance imaging and corresponding magnifications show bilateral hyperintense lesions, indicating acute ischemic cerebral injury. **(B)** Quantifying the amount of diffusion restriction, the apparent diffusion coefficient map indicates ischemic lesions. The arrows indicate the corresponding lesions.

To address her prolonged impaired consciousness and ongoing focal epileptic seizures, a brain magnetic resonance imaging (MRI) scan on day 3 after admission showed bilateral areas of restricted diffusion in the diffusion-weighted imaging (DWI) and apparent diffusion coefficient (ADC) maps indicating ischemic lesions (Figure 2). Up to this point, laboratory diagnostics revealed elevated troponin enzymes (404 pg/ml, normal range: <24 pg/ml) and elevated creatine kinase muscle-brain subunit levels (1,563 pg/ml, normal range: <170 pg/ml). A 12-lead electrocardiogram (ECG) and continuous monitoring showed no ST elevation, no arrhythmia though corrected QT time was prolonged with 560 ms. Subsequently, transesophageal echocardiography (TEE) was performed and showed no signs of endocarditis, ventricular dysfunction, or thrombogenic material. No prior cardiovascular risk factors could be determined. She was normotensive and non-diabetic (HbA1c: 5.2%), with a normal lipid profile (HDL, LDL, LpA, and LDL/HDL ratio), a normal body mass index (BMI: 20 kg/m^2^), and a negative family history for cardiovascular events. Basic laboratory diagnostics revealed normal electrolytes (e.g., Na^+^, K^+^) and blood count range. Furthermore, an extended stroke workup, including long-term ECG, transcranial Doppler sonography, and serological tests for thrombophilia, including genetic mutations, showed no abnormalities.

While treating our patient, we were faced with several different diagnostic challenges. Two days after the admission, we learned about the patient's borderline personality disorder diagnosis and her prescribed medication, venlafaxine, through external anamnesis. Subsequently, 3 days after admission, toxicological screen results revealed venlafaxine intoxication, which led to our working hypothesis of serotonin syndrome.

Retrospectively, our patient demonstrated many features of serotonergic syndrome resulting in disturbances of consciousness and memory (e.g., confusion, disorientation, and impaired consciousness), disturbances of the autonomic nervous system (e.g., hyperhidrosis, hyperthermia, and tachycardia), and neuromuscular abnormalities (e.g., epileptic seizures). Initially, competitive etiologies for the combination of unstable vital parameters, impaired consciousness, and focal neurologic complications, such as, intracerebral bleeding, intracerebral tumor, intra- or extracranial dissection, and extracranial bleeding, were ruled out using polytrauma computed tomography. Infectious intracerebral diseases, including meningitis and/or encephalitis, were ruled out using lumbar puncture. The combination of elevated troponin enzymes, bilateral ischemic strokes, unstable vital parameters, and possibly unknown interacting additional intravenous drug abuse resulted in the differential diagnosis of endocarditis and/or myocarditis, which were ruled out using consecutive blood cultures and TEE. Competing differential diagnosis for ischemic strokes in young patients, such as extra- or intracranial dissection of vessels, as well as genetic disorders for thrombophilia or autoimmune diagnosis cumulating in vasculitis, were additionally ruled out using time-of-flight (TOF) imaging and laboratory tests. Considering the contributing severe and prolonged hypoglycemia, both an insulinoma and external abuse of insulin were discussed; however, they were excluded due to the abdominal sonography and by measuring C-peptide concentrations. Because our patient did not suffer from headaches and typical migraine headaches in the past, a causal connection to our patient cannot be made, although migraine without headaches might explain some of the symptoms.

Initially, the hypoglycemia was treated with glucose 40% solution intravenously; however, our patient needed repeated glucose infusions (total: 600 ml glucose 40% solution) during the first 3 days. Additionally, fluid in the form of Sterofundin ISO 2,000 ml/day was administered symptomatically. Epileptic seizures had been addressed with short-acting diazepam 10 mg and further dose escalation using levetiracetam 1,000 mg twice daily. The patient's body temperature was monitored every 4 h, and we observed a normalization within the 1^st^ day of admission (Figure 1B).

The patient was discharged to the psychiatric facility with no remaining neurologic deficit. Regarding the ischemic stroke, secondary prophylaxis with thrombocyte-inhibiting agents and statins was not started because our patient showed normotensive blood pressure values, a normal lipid status, no genetic alterations, and no evidence of atrial fibrillation or micro- or macroangiopathic genesis. No follow-up was planned. No adverse or unanticipated events took place.

## Discussion

Since venlafaxine was introduced to the market in the 1990s, its use has increased, and it has often been prescribed to patients who have not responded to selective serotonin reuptake inhibitors or tricyclic antidepressants (Rubino et al., 2007). Recent studies have reported that these patients, suffering from more severe and treatment-resistant depression, presented a higher risk of dying by suicide or attempting suicide (Rubino et al., 2007). Recent concerns about cardiovascular safety have even led to restrictions in prescribing venlafaxine in the United Kingdom (Howell et al., 2007). We report a case of a 21-year-old female patient with no prominent cardiovascular risk factors who suffered from bilateral ischemic strokes, prolonged and glucose-resistant hypoglycemia, seizures, and myocardial injury, most likely due to venlafaxine intoxication.

Our patient demonstrated many features of serotonergic syndrome resulting in disturbances of consciousness and memory, disturbances of the autonomic nervous system, and neuromuscular abnormalities. The underlying mechanisms of cerebral infarctions in venlafaxine intoxication are poorly understood; however, a mechanism underlying serotonin syndrome that causes diffuse vasospasm and microvascular dysfunction has been described (Vasudev et al., 2016). To our knowledge, no cases of venlafaxine-intoxication-induced cerebral ischemia have been reported. Nonetheless, reversible segmental cerebral vasoconstrictions, which are associated with serotonergic agents such as paroxetine and venlafaxine, have been reported. This so-called Call–Flemings syndrome is associated with acute thunderclap headache, seizures, and focal neurologic deficits, caused by a diffuse vasoconstriction of cerebral vessels (Gilbert, 2002; Noskin et al., 2006). In our case, no acute onset of headache was described, and no segmental cerebral vasoconstrictions were detected in the transcranial Doppler sonography. Apart from an occasional abuse of cigarettes and cannabinoids, no cardiovascular risk factor could be determined and additional intravenously abuse of serotonergic or adrenergic drugs could be ruled out. Competing possible cardioembolic etiologies for bilateral cortical strokes in our patient, for example, endocarditis, could be ruled out, even though her plasma hs-troponin levels were elevated. The ischemic lesions found in the MRI scan were very small and, apart from an altered state of consciousness, caused no neurological deficit; nonetheless, considering the number of people using venlafaxine and the potential for misuse, the clinical impact of these lesions is prominent.

Our patient developed several focal epileptic seizures with tonic-clonic contractions. We propose that a combination of prolonged hypoglycemia, serotonergic effects, and adrenergic side effects contributed to hyperexcitability. Recent studies have shown that venlafaxine is pro-convulsive due to a rate-dependent effect on sodium channels at micromolar concentrations (Khalifa and Daleau, 1999). As described in an earlier case report, lamotrigine should not be used to treat seizures in patients with venlafaxine intoxication, due to its pro-arrhythmic effects; therefore, benzodiazepines and levetiracetam were used (Howell et al., 2007).

Prolonged and glucose-resistant hypoglycemia is a common adverse event following treatment with antidepressants, especially in antidepressants with a high affinity for serotonin reuptake (Özdemir, 2022; Derijks et al., 2008). However, the underlying mechanism causing hypoglycemia in patients with venlafaxine overdose is poorly understood. Cases of both disturbances of insulin levels and insulin-independent hypoglycemia are described (Francino et al., 2012). In this case, plasma insulin, pro-insulin, and C-peptide levels were in normal ranges during episodes of hypoglycemia and food restriction, indicating no participation of an insulinoma. The prolonged, glucose-resistant hypoglycemia might be explained by an increase of glucose uptake in peripheral tissues due to both serotonin-induced beta-endorphin release and stimulation of increased muscle glucose utilization, which has been described in the literature (Brvar et al., 2015). Another reason for the prolonged hypoglycemia in our patient might be due to enhanced opioid pathway stimulation via the mu-opioid receptor because venlafaxine is similar in structure to tramadol, which is known to cause hypoglycemia (Bourne et al., 2013).

Prolonged hypoglycemia might also be due to gastric bezoar formation, following venlafaxine overdose and the continuous, delayed release of venlafaxine; therefore, we would recommend performing an endoscopic examination of the esophagus, stomach, and duodenum (Lung et al., 2011; Djogovic et al., 2007). Given that long-term and therapy-resistant hypoglycemia may worsen the initial cerebral injury, we recommend promptly addressing the hypoglycemia and closely monitoring blood glucose levels.

In association with our case reports and our hypothesis of a venlafaxine-induced serotonin syndrome cumulating in diffuse vasospasm in cerebral and myocardial arteries, several limitations must be considered. In this case, we had the unique opportunity to present a young patient with no confounding micro- or macro-cardiovascular risk factors, aside from borderline spectrum disorder; no contributing autoimmune or systemic diseases; and, apart from venlafaxine, no interfering co-medication. We therefore hypothesize that many, if not all, of the side effects presented in this case report can be attributed to the venlafaxine intoxication itself, rather than underlying cardiovascular diseases.

However, due to our patient's unstable conditions, impaired consciousness in the early phase, and delayed anamnesis, there was a 3-day delay between toxicology screen results and admission, which made investigating the very early stages of venlafaxine intoxication impossible. Consequently, the MRI scan was performed on day 3 after admission, and because irreversible (match in DWI/ADC) acute ischemic cerebral injuries were detected, no additional invasive/non-invasive angiographic study was made in the TOF sequences; however, no signs of vasospasm were detected. On day 4 after admission, transcranial Doppler sonography did not detect any segmental cerebral vasoconstrictions. Nevertheless, we hypothesize an early mechanism of cerebral vasoconstriction due to the adrenergic side effects of venlafaxine, which we were unable to detect following the long delay between the initial admission and the transcranial Doppler sonography. An alternative etiology of the detected DWI/ADC MRI abnormalities might be prolonged epileptic seizures.

## Conclusion

We presented a case of a patient suffering from cerebral strokes and myocardial injury due to venlafaxine intoxication. We hypothesized a venlafaxine-induced serotonin syndrome cumulating in diffuse vasospasm in cerebral and myocardial arteries due to an autonomic effect mediated by the serotonergic and adrenergic systems. Collectively, our study provides novel findings and presents an important adverse event following venlafaxine intoxication, which readers should keep in mind when examining patients with impaired consciousness, hypoglycemia, epileptic seizures, and prescribed serotonergic drugs (e.g., venlafaxine). Furthermore, this case report proposes many important diagnostic and therapeutic aspects for treating the causal serotonin syndrome. Hypoglycemia and fever should be treated symptomatically. Epileptic seizures should be treated using short-acting benzodiazepines or levetiracetam; lamotrigine should not be used due to its pro-arrhythmic effects. A gastrointestinal endoscopic examination should be considered to rule out bezoar formation, which might maintain hypoglycemia and epileptic seizures, caused by the continuous release of venlafaxine (Djogovic et al., 2007). These findings might have important implications at the level of basic clinical considerations not only for general practitioners but also, predominantly, for psychiatrists and neurologists.

After 5 days of intermediate care, our patient was discharged to the psychiatric facility with no remaining neurological deficits. Our patient was informed about the possible risks and consequences of venlafaxine intoxication and agreed to undergo further psychiatric inpatient treatment.

## Data Availability

The original contributions presented in the study are included in the article/supplementary material, further inquiries can be directed to the corresponding author.
